# TET3- and OGT-Dependent Expression of Genes Involved in Epithelial-Mesenchymal Transition in Endometrial Cancer

**DOI:** 10.3390/ijms222413239

**Published:** 2021-12-08

**Authors:** Piotr Ciesielski, Paweł Jóźwiak, Ewa Forma, Anna Krześlak

**Affiliations:** Department of Cytobiochemistry, Faculty of Biology and Environmental Protection, University of Lodz, 90-236 Łódź, Poland; piotr.ciesielski@biol.uni.lodz.pl (P.C.); pawel.jozwiak@biol.uni.lodz.pl (P.J.); ewa.forma@biol.uni.lodz.pl (E.F.)

**Keywords:** endometrial cancer, TET proteins, O-GlcNAc transferase, *TWIST1*, *ZEB1*, *FOXC1*, migration, invasion

## Abstract

TET3 is a member of the TET (ten-eleven translocation) proteins family that catalyzes the conversion of the 5-methylcytosine into 5-hydroxymethylcytosine. TET proteins can also affect chromatin modifications and gene expression independently of their enzymatic activity via interactions with other proteins. O-GlcNAc transferase (OGT), the enzyme responsible for modification of proteins via binding of *N*-acetylglucosamine residues, is one of the proteins whose action may be dependent on TET3. Here, we demonstrated that in endometrial cancer cells both TET3 and OGT affected the expression of genes involved in epithelial to mesenchymal transition (EMT), i.e., *FOXC1*, *TWIST1*, and *ZEB1*. OGT overexpression was caused by an increase in *TWIST1* and *ZEB1* levels in HEC-1A and Ishikawa cells, which was associated with increased O-GlcNAcylation of histone H2B and trimethylation of H3K4. The TET3 had the opposite effect on gene expressions and histone modifications. OGT and TET3 differently affected *FOXC1* expression and the migratory potential of HEC-1A and Ishikawa cells. Analysis of gene expressions in cancer tissue samples from endometrial cancer patients confirmed the association between OGT or TET3 and EMT genes. Our results contribute to the knowledge of the role of the TET3/OGT relationship in the complex mechanism supporting endometrial cancer progression.

## 1. Introduction

TET family proteins play a significant role in changing the pattern of DNA methylation through participation in DNA demethylation. In humans, three TET proteins named TET1, TET2, and TET3 have been identified. These proteins are iron(II)- and 2-ketoglutarate-dependent dioxygenases, which catalyze the conversion of the 5-methylcytosine (5-mC) into 5-hydroxymethylcytosine (5-hmC) and further into 5-formylcytosine (5-fC) and 5-carboxycytosine (5-caC), which initiates the process of DNA demethylation [1,2,3]. Besides being involved in DNA demethylation, TET proteins can also affect the epigenetic modifications, regardless of their enzymatic activity by interacting with other proteins that are involved in the modification of chromatin, for example, O-GlcNAc transferase (OGT) [4,5,6]. OGT is an enzyme responsible for the modification (O-GlcNAcylation) of cellular proteins by linking the single *N*-acetylglucosamine moieties to serine or threonine residues by the O-glycosidic bond. This dynamic modification affects the activity and stability of a great number of proteins, including metabolic enzymes, kinases, phosphatases, transcription factors, and many others. Increased expression of OGT and hyper-O-GlcNAcylation are the hallmarks of many tumors [7,8,9]. It has been found that the O-GlcNAcylation may be a part of histone code, and OGT modifies H2A, H2B, H3, and H4 histones [10]. Studies using chromatin immunoprecipitation (ChIP-Seq) showed that TET1, TET2, TET3, and OGT can colocalize in H3K4me3-rich sites of chromatin in promoters of transcriptionally active genes [10,11].

It is suggested that TET proteins play an essential role in the recruitment of OGT to chromatin so that it can modify histones [4,5,12]. Chen et al. [4] demonstrated that in murine embryonic stem cells (mESC), Tet2 protein is necessary for OGT to modify histone H2B at serine 112 (H2BS112GlcNAc). Glycosylation of this serine residue facilitates the further modification of histone H2B, the monoubiquitination of lysine 120 (H2BK120Ub). Studies of Ito et al. [5] indicated that TET3 plays the main role in the recruitment of O-GlcNAc transferase to chromatin and regulates its stability.

The epithelial to mesenchymal transition (EMT) is a biological process in which epithelial cells experience profound changes in motility and their ability to invade surrounding tissues [13]. Major players in the regulation of EMT are transcription factors, designated as EMT-TFs, which include, among others, Zinc finger E-box-binding homeobox 1 and 2 (ZEB1 and ZEB2), and Twist-related protein 1 and 2 (TWIST1 and TWIST2). These factors take part in a complex network that activates a specific molecular program aimed at repressing epithelial markers (e.g., E-cadherin, *CDH1*) and activating mesenchymal markers (e.g., vimentin, *VIM*) [13].

The pioneer factors, a special class of transcription factors that can associate with compacted chromatin to facilitate the binding of additional transcription factors are also involved in cancer progression. The function of pioneer factors was originally described during development. More recently, they have been implicated, especially FOXA1, in hormone-dependent cancers, such as estrogen receptor-positive breast cancer and androgen receptor-positive prostate cancer [14]. The role of FOXA1 in human malignancy remains incompletely defined, as both pro- and antitumorigenic functions have been uncovered. FOXA1 is strongly associated with metastatic disease in prostatic adenocarcinoma [15]. In breast cancer, high FOXA1 expression positively correlates with the outcome, but the potential impact of its expression depends on the ERα status and tumor molecular subtype [15]. The other important factor whose role in cancer development and progression has begun to emerge is FOXC1 [16]. Although FOXC1 has not been formally confirmed as a pioneer factor it seems probable it is one since it exhibits conservation of the critical amino acids which confer pioneer activity in FOXA1. Overexpression of FOXC1 has been reported in at least 16 types of cancer, often in association with a poor prognosis [16].

Here, we demonstrate that OGT and TET3 affect the expression of genes associated with epithelial-mesenchymal transition via changes of histones modifications and the ability of endometrial cancer cells for migration and invasion.

## 2. Results

### 2.1. Effect of OGT and TET3 on the Expression of Genes Involved in EMT

**Figure 1:**
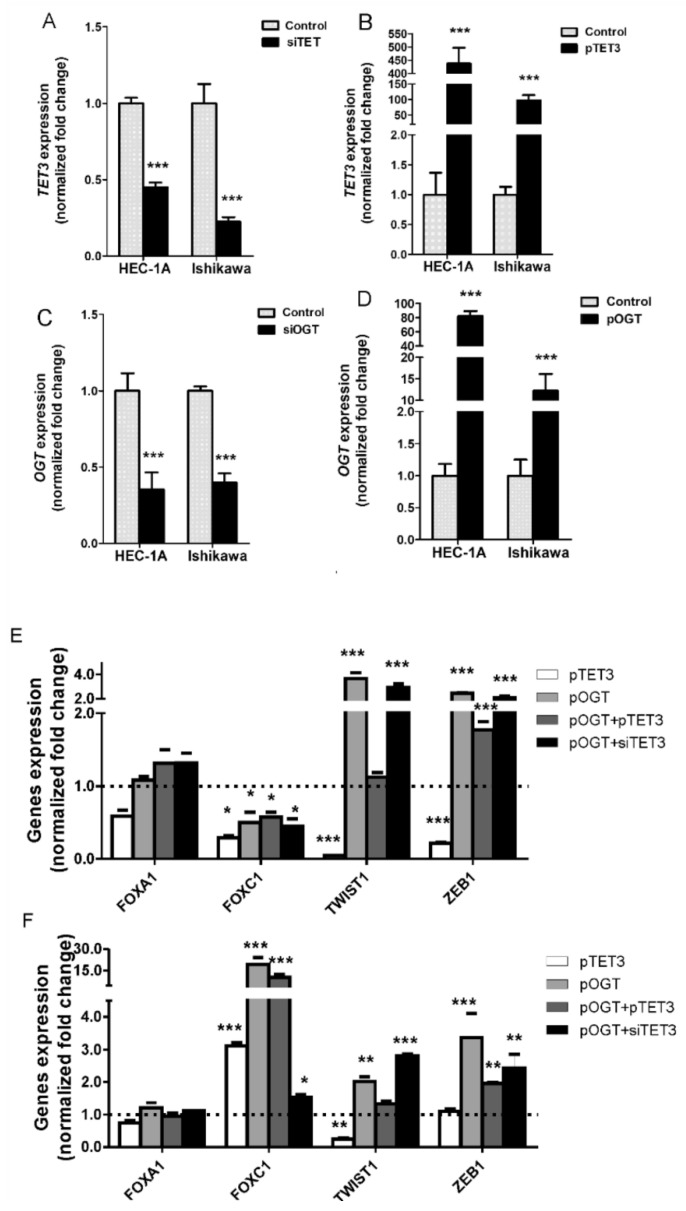
Gene expressions in cells with TET3 and OGT dysregulation. In HEC-1A and Ishikawa cells, TET3 (**A**) and OGT (**C**) expression were decreased by transfection of cells with specific siRNA or increased via transfection with plasmid DNA (**B**,**D**) respectively). The mRNA levels were analyzed by real-time PCR methods (for details see Methods). The mRNA expression of *FOXA1*, *FOXC1*, *TWIST1*, and *ZEB1* were evaluated in HEC-1A (**E**) and Ishikawa (**F**) cells with increased expression of TET3 (pTET3), OGT (pOGT), co-expression of OGT and TET3 (pOGT + pTET3), and increased expression of OGT and reduced expression of TET3 (pOGT + siTET3). The expressions of genes in each kind of sample were compared to appropriate controls, i.e., cells treated with empty vectors or nonsilent siRNA duplexes in which expression was assumed to be 1. Data show mean ± SE (*n* = 5), * *p* < 0.01, ** *p* < 0.001, *** *p* < 0.0001.

The impact of changes in OGT and TET3 amounts on the expression of genes involved in epithelial-mesenchymal transition (*FOXA1*, *FOXC1*, *TWIST*, *ZEB1*) in endometrial cancer cells has been analyzed. Expressions of TET3 and OGT were changed by treating HEC-1A and Ishikawa cells with plasmid vectors or siRNA (Figure 1A–D). In endometrial cancer cells with TET3 and OGT dysregulation, the expressions of *FOXA1, FOXC1, TWIST, ZEB1* were analyzed and the results are shown for HEC-1A and Ishikawa cells in Figure 1E,F, respectively. The expressions were analyzed in cells with unchanged OGT and TET3 (control), cells with overexpression of TET3 (pTET3), cells with overexpression OGT (pOGT), and cells with overexpression both OGT and TET3 (pTET3/pOGT), and cells cotransfected with pOGT and siTET3. Changes in expression of TET3 and OGT affected the expression of *FOXC1*, *TWIST1*, and *ZEB1* but did not significantly influence the expression of *FOXA1*. Overexpression of TET3 and OGT caused decreased expression of *FOXC1* in HEC1A cells and increased expression of this gene in Ishikawa cells. TET3 and OGT seem to have the opposite effect on *TWIST1* expressions both in HEC1A and Ishikawa cells, i.e., TET3 causes decreased expression and OGT causes increased expression. Expression of *ZEB1* was affected mostly by OGT, especially in Ishikawa cells. When TET3 and OGT were coexpressed, the most interesting results were found for *TWIST1*. TET3 seems to counteract the *TWIST1* expression increase caused by OGT. In the case of *ZEB1*, co-overexpression of OGT and TET3 give the same results as overexpression of OGT alone. To confirm the results of the TET3 and OGT impact on these genes, we also analyzed the expression of genes in cells treated with siRNAs specific for TET3 or OGT. The results are shown in Supplementary Figure S1. These results support the findings from overexpression experiments.

### 2.2. Amount and Localization of OGT in Endometrial Cancer Cells with TET3 Overexpression

**Figure 2:**
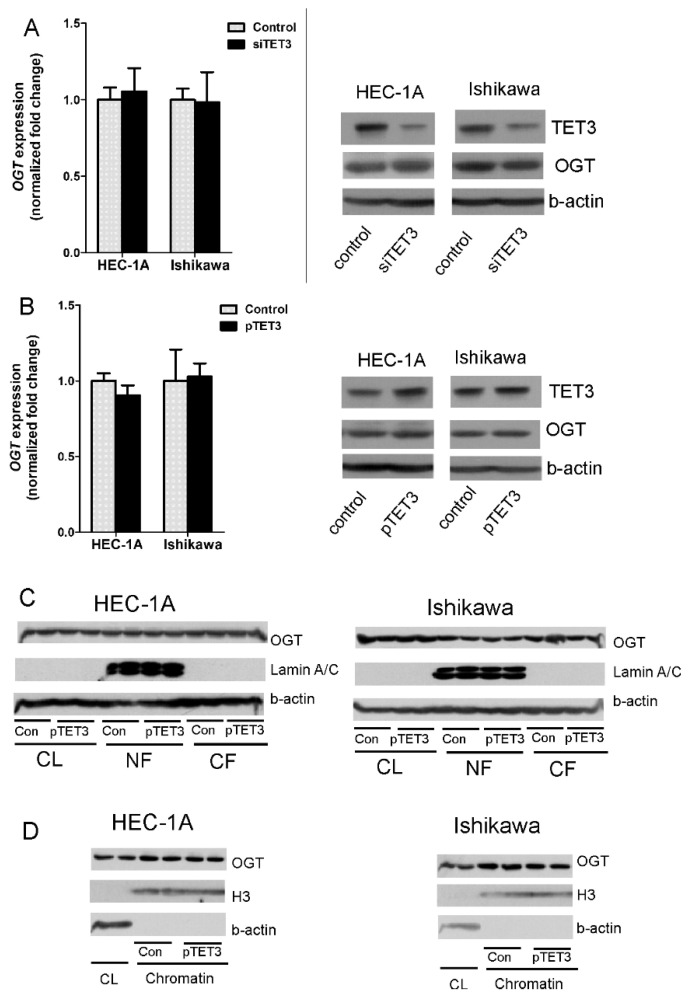
OGT expression and localization in cells with TET3 deregulation. OGT expression was analyzed at mRNA and protein levels in HEC-1A and Ishikawa cells with TET3 reduced (**A**) or increased (**B**) expression using the real-time PCR method or Western blot method. (**C**,**D**) are the results of OGT identification by Western blot method in cellular fractions in control cells and cells with TET3 overexpression. CL—whole cell lysate; NF—nuclear fraction; CF—cytoplasmic fraction.

It has been suggested that interactions between TET3 and OGT may impact the stability and intracellular localization of OGT [5]. Thus, the impact of TET3 dysregulation on the expression and localization of OGT in chromatin fraction in endometrial cells was analyzed. In HEC-1A and Ishikawa cells treated with TET3 siRNA or plasmid DNA, the OGT expression was analyzed. The mRNA and protein levels of OGT in cells with TET3 down- and up-regulation did not change (Figure 2A,B). TET3 overexpression did not affect the localization of OGT as well. There was no difference in the global cytoplasmic and nuclear amount of OGT between control cells and cells overexpressing TET3 (Figure 2C). Control cells and cells treated with transcription vector were also fractionated to obtain the chromatin fraction. The results showed enrichment of OGT in chromatin fraction, but TET3 overexpression did not impact OGT localization in chromatin fraction (Figure 2D).

### 2.3. Impact of TET3 and OGT on Each Other Binding to Chromatin and Histones Modifications 

**Figure 3:**
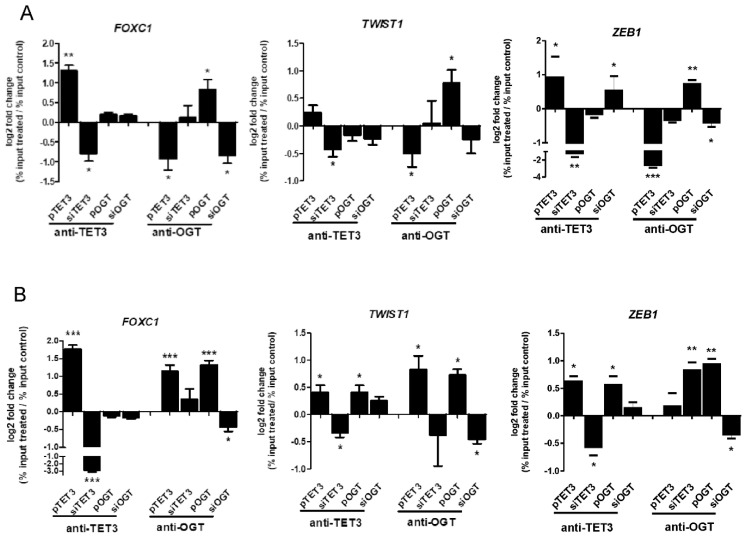
OGT and TET3 binding to chromatin in *FOXC1*, *TWIST1*, and *ZEB1* regions. Chromatin immunoprecipitation (ChIP) analysis was performed for HEC-1A (**A**) and Ishikawa (**B**) endometrial cancer cells treated with plasmid DNA or siRNA with specific antibodies. Differential chromatin enrichment was quantified using real-time quantitative PCR. The results were calculated as % input (% input = % input of specific antibodies probes—% input mock IgG) and are presented as a log2fold change (Fold change = % input of treated cells sample/% input of control cells sample). Data show mean ± SE, (*n* = 4) * *p* < 0.01, ** *p* < 0.001, *** *p* < 0.0001.

Since both TET3 and OGT deregulation had a significant impact on *FOXC1*, *TWIST*, and *ZEB1* expressions, the binding of TET3 and OGT proteins to chromatin associated with these genes were analyzed. The results showed that both TET3 and OGT bound to chromatin in regions of EMT genes in HEC-1A and Ishikawa cells (Figure 3A,B, respectively). Overexpression of TET3 or OGT in both cells caused increased and siRNA decreased binding to chromatin compared to appropriate control cells. The effect of overexpression or downregulation of OGT and TET3 on each other’s amounts in chromatin associated with *FOXC1*, *TWIST1*, and *ZEB1* was analyzed. In HEC-1A cells, the OGT changed significantly the anti-TET3 antibody binding only in the case of *ZEB1*, but TET3 overexpression reduced the anti-OGT antibody binding to chromatin in regions of all three genes. TET3 overexpression in Ishikawa cells was associated with increased binding of the anti-OGT antibody to the region of *FOXC1* and *TWIST1*.

**Figure 4:**
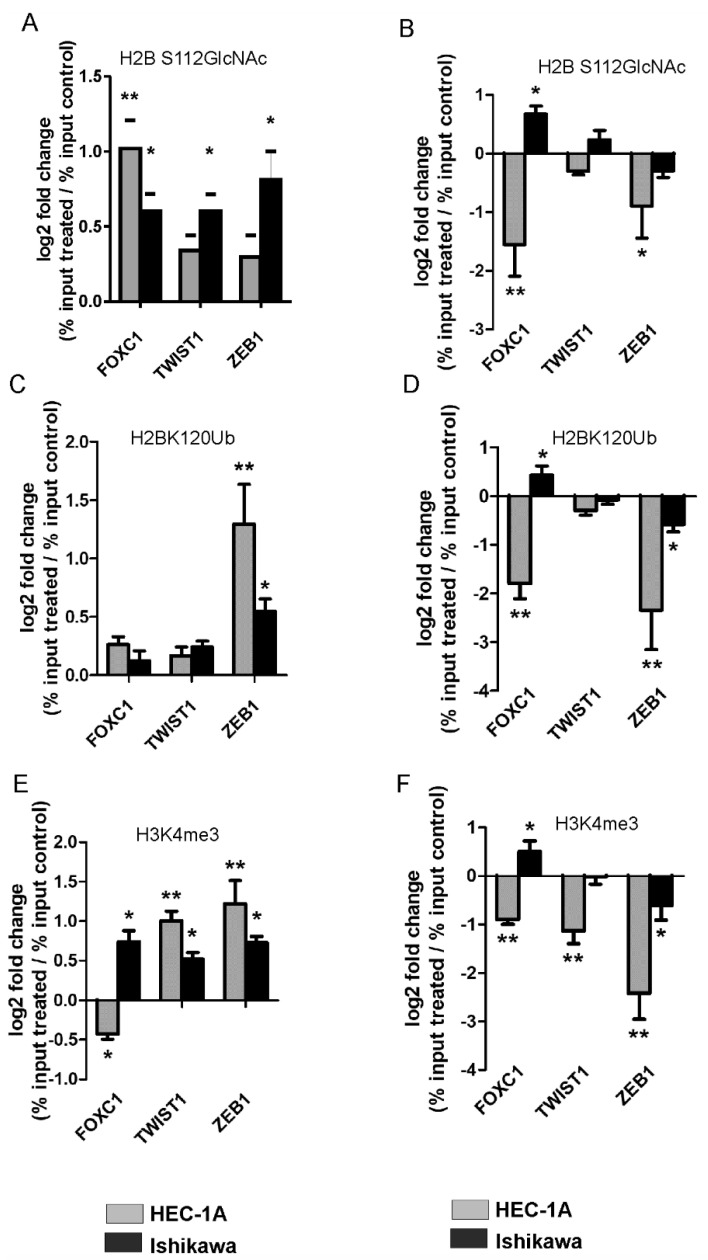
OGT and TET3 dysregulation affect histone modifications. The O-GlcNAcylation of serine 112 of histone H2B (H2BS112GlcNAc), ubiquitination of lysine 120 of histone H2B (H2BK120Ub), and trimethylation of lysine 4 of histone H3 (H3K4me3) have been analyzed using the ChiP method in cells with OGT (**A**,**C**,**E**) or TET3 (**B**,**D**,**F**) overexpression. Differential chromatin enrichment was quantified using Real-time quantitative PCR. The results were calculated as % input (% input = % input of specific antibodies probes—% input mock IgG) and are presented as a log2fold change (Fold change = % input of treated cells sample/% input of control cells sample). Data show mean ± SE, (*n* = 5) * *p* < 0.01, ** *p* < 0.001.

The modifications of histones were analyzed in cells with overexpression of OGT or TET3 (Figure 4). Generally, overexpression of OGT caused increased O-GlcNAcylation of histone H2B and trimethylation of lysine 4 of histone H3 (Figure 4A,D). The only exception was the *FOXC1* region where methylation was decreased after OGT overexpression. The overexpression of TET3 caused generally decreased O-GlcNAcylation and ubiquitination of histone H2B and methylation of histone H3 in the case of all analyzed genes (Figure 4B,F). However, there was also an exception. In Ishikawa cells overexpression of TET3 caused increased O-GlcNAcylation, methylation, and ubiquitination of histones in chromatin in the *FOXC1* region. Thus, OGT and TET3 had a similar effect on the methylation of H3K4 in HEC-1A and Ishikawa cells. Increased methylation of H3K4 was associated with increased expression of *FOXC1*.

### 2.4. OGT and TET3 Affect Migration and Invasion of Endometrial Cancer Cells

**Figure 5:**
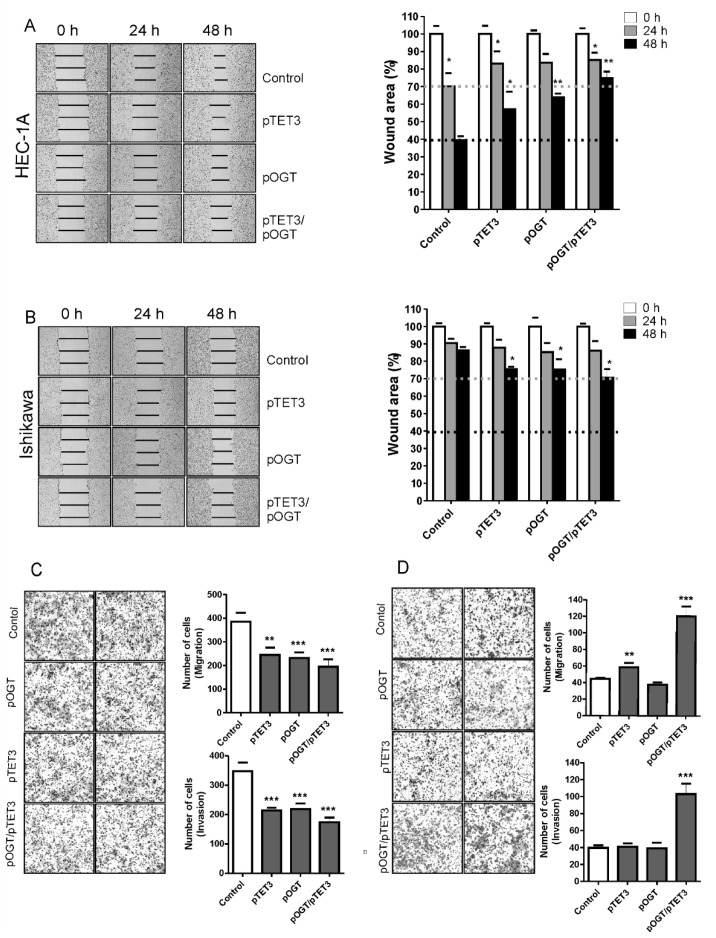
Effect of OGT and TET3 on migration and invasion of cells. The wound-healing assay was performed to examine the migration rate of HEC-1A (**A**) and Ishikawa cells (**B**) transduced with OGT and TET3 plasmid vectors. Photographs were taken at 0, 24 h, and 48 h following the initial scratch. Migration rates were quantified by measuring three different wound areas. Three separate experiments were performed. Cell migration assays using Transwell chambers and invasion assays using Transwell chambers with Matrigel were performed for HEC-1A (**C**) and Ishikawa cells (**D**). Representative images of migrating cells stained with Giemsa are displayed (left) for HEC-1A (**C**) and Ishikawa cells (**D**). Quantitative data of migration and invasion assay are expressed relative to the migration and invasion abilities of control cells. Plots show average counts from three independent testings * *p* < 0.01; ** *p* < 0.001; *** *p* < 0.0001.

Wound healing and Boyden chamber assays were conducted to evaluate the migration and invasion capacity of endometrial cancer cells with overexpression of OGT and TET3 (Figure 5). Interestingly, the effect of TET3 and OGT overexpression was opposite in HEC-1A and Ishikawa cells. In HEC-1A cells, the overexpression of TET3 and OGT caused decreased migration and invasion. The migration potential of Ishikawa control cells was very small, and it was slightly increased after OGT or TET3 expression. The results of Boyden chamber assays showed that both migration and invasion of cells were significantly increased after coexpression of OGT and TET3.

### 2.5. Correlations between Expression of EMT Genes and OGT or TET3 in Endometrial Cancer

**Figure 6:**
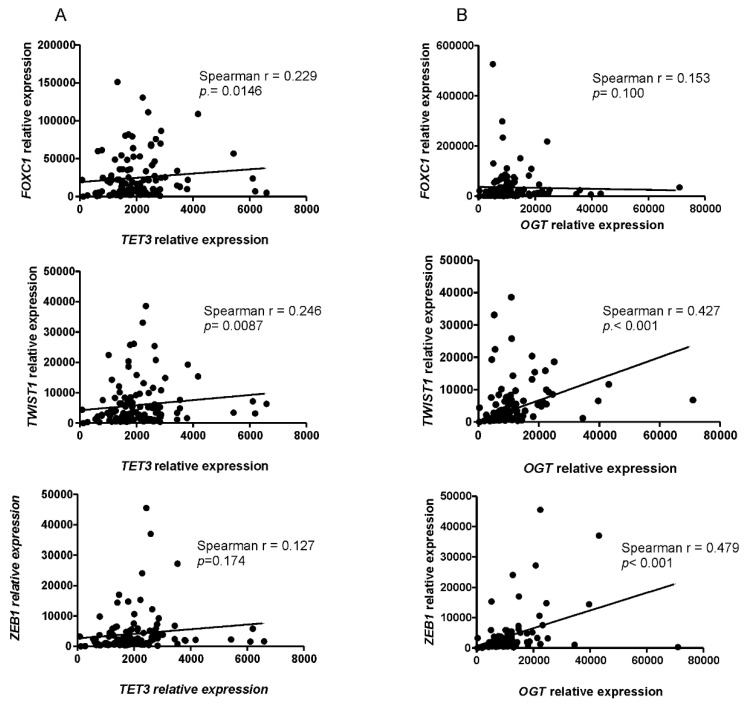
Correlations between gene expressions in endometrial cancer samples. Expression levels of TET3, *OGT*, *FOXC1*, *TWIST1*, and *ZEB1* in samples of endometrial cancers were evaluated by real-time quantitative PCR analysis with the *HPRT1* gene applied as a reference. Spearman rank correlation analysis was performed to analyze the association of TET3 (**A**) or OGT (**B**) expression with *FOXC1*, *TWIST1*, and *ZEB1*. The correlations were considered significant when *p* < 0.05.

To further investigate whether OGT and TET3 were associated with the expression of EMT genes in endometrial cancer, we analyzed the correlations between these gene expressions in 131 samples of endometrial cancer tissues using quantitative PCR. The results showed a significant moderate correlation between OGT expression and *TWIST1* (r = 0.427) or *ZEB1* (r = 0.479) (Figure 6). There was also a weak correlation between TET3 and *FOXC1* (r = 0.229) and *TWIST1* (r = 0.247) (Figure 6).

## 3. Discussion

Most earlier studies of TET proteins focused on their ability to facilitate DNA demethylation through the production of 5-hmC. It has only recently been recognized that TET proteins can also affect chromatin modifications and gene expressions independently of their enzymatic activity. It is suggested that individual TET proteins via interactions with other proteins may indirectly change the expressions of specific genes [17]. Moreover, the individual TET proteins may have a different impact on cancer onset and progression. TET1 decreased expression and low 5-hmC levels are frequently observed in many different types of cancers, including gastric, prostate, liver, lung, and breast cancer as well as glioblastoma and melanoma [18]. The TET2 gene is subjected to frequent somatic mutations in an extensive range of hematopoietic cancers, including myeloid and lymphoid cancers and several solid cancers [19]. In contrast to decreased expression of TET1/2 in cancers, it was found that TET3 expression was upregulated in ovarian cancer, and its high expression was correlated with poor clinicopathological features [20]. Our previous studies showed that TET1 and TET2 messenger RNA expression was lower and TET3 expression was higher in endometrial cancers compared to normal tissues [21]. A positive correlation between 5-hmC and the relative expression of TET1 and TET2 was found, but no correlation was observed in the case of TET3 [21]. Thus, the role of TET3 in endometrial cancer seems to be different than TET1 or TET2, and this protein may be involved in modifications of histones by targeting other proteins to chromatin. It has been suggested that O-GlcNAc transferase is one of the TET3 partners [5].

In this study, we analyzed the impact of TET3 and OGT on migration and invasion of endometrial cancer cells via regulation of EMT genes expression. Our results show that both TET3 and OGT affect cell migration and invasion of endometrial cancer cells; however, unexpectedly, their effects are different in HEC-1A and Ishikawa cells. Ishikawa cells are well-differentiated and their migratory potential is low. TET3 and OGT overexpression caused an increase in the migratory potential of Ishikawa cells, which was especially seen when both proteins were coexpressed. Increased expression of TET3 and OGT caused a significant increase of *FOXC1* expression. Interestingly, increased expression of TET3 caused increased binding of OGT to chromatin in the region of *FOXC1* and increased histone H2B O-GlcNAcylation and H3K4 trimethylation. This may suggest that TET3 plays a role in the targeting of OGT to chromatin in Ishikawa cells. Although our results did not show general enrichment of OGT in chromatin fraction after TET3 overexpression, that did not exclude the possibility that TET3 is involved in the targeting of OGT to specific chromatin sites. HEC-1A cells, contrary to Ishikawa, showed decreased migratory potential after TET3 and OGT overexpression. Interestingly, in these cells, *FOXC1* expression was decreased after TET3 or OGT overexpression. We cannot explain the reason for different TET and OGT impacts on *FOXC1* in the two cell lines. Both cell types represent type I endometrial cancer. Traditionally, endometrial cancer has been divided into two subtypes with distinct clinical, pathological, histological, and molecular behavior. Type I endometrial carcinomas account for 85% of all endometrial cancers, and they are mainly low grade, estrogen-dependent, hormone-receptor-positive adenocarcinomas with endometrioid morphology [22]. However, despite many similarities, these two types of cells differ in many ways. For example, Ishikawa cells do not express *PTEN*, and HEC-1A cells are *PTEN*-wild-type. On the other hand, Ishikawa cells expressed all estrogen receptors, and HEC-1A cells lack expression of *ESR1*. Ishikawa cells form more estradiol from estrone than HEC-1A cells [23]. The relationship between ER α and the expression of EMT genes is well established for breast cancer [24]. Thus, we think that the different molecular background lies behind the different effects of TET3 and OGT on *FOXC1*. Future studies are necessary to identify all TET and OGT partners involved in FOXC1 regulation. Although the impact of TET3 and OGT on *FOXC1* expression in HEC-1A and Ishikawa are opposite, it seems that endometrial cell migration and invasion are correlated with this factor expression. Several studies have linked *FOXC1* activity to the aggressive phenotype in cancer cells, especially in basal-like breast cancer and hepatocellular carcinoma [25]. Although studies of FOXC1 in endometrial cancer are not advanced, it is suggested that *FOXC1* may be a potential oncogene also in endometrial carcinoma [25]. In endometrial cancer, the downregulation of *FOXC1* by miRNA—specifically miRNA 204 and miRNA 495—caused inhibition of cancer cell growth and migration [26,27]. The results of our research seem to confirm the significance of *FOXC1* in the aggressive phenotype of endometrial cancer cells. However, the analysis of mRNA expression of FOXC1 in endometrial cancer tissues did not show any association of *FOXC1* expression with clinicopathological characteristics (Supplemental Figure S2).

The findings of this study suggest that OGT and TET have the opposite effect on *TWIST1* and *ZEB1* expressions and histone modifications, both in HEC-1A and Ishikawa cells. OGT increases of O-GlcNAcylation of H4S112 and methylation of H3K4 in the ZEB1 region, which results in the increased expression of *ZEB1*. On the contrary, TET3 overexpression was associated with decreased O-GlcNAcylation and methylation. OGT overexpression also significantly increased H3K4 methylation and expression of *TWIST1*, while TET3 reduced both methylation and expression. However, the migratory potential of HEC-1A cells was not correlated with the increased expression of *ZEB1* or *TWIST1*. The previous studies of Feng et al. [28] showed that *ZEB1* was related to the metastasis of endometrial cancer. *ZEB1* expression was significantly associated with subtype, grade, myometrial invasion, and lymph node metastases in endometrial cancer [28]. Similarly, Shen et al. [29] also showed significantly higher *TWIST1* expression in patients with type I endometrial cancer compared to normal endometrium, and aberrant *TWIST1* expression was significantly associated with clinical parameters, indicating poor prognosis and shorter patient survival. However, Sadłecki et al. [30] did not find significant associations between the clinicopathological characteristics of endometrial cancer patients and the expressions of TWIST1, TWIST2, ZEB1, and *SNAIL*. Our results also did not show any correlation between TWIST1 expression and clinicopathological parameters and *ZEB1* expression was even lower in more advanced cancers compared to less aggressive cancers (Supplementary Figures S3 and S4).

Thus, the function of EMT inducers in endometrial cancers needs further explanation. The expression and function of EMT inducers may vary considerably across different cancer types or even cell types depending on the molecular context [31]. For example, *TWIST1* was found to promote metastasis in the breast cancer model but was shown to be dispensable for metastasis in a pancreatic cancer model. Sometimes, factors may have even opposite effects in different tumors. *ZEB2* is associated with metastasis in ovarian, gastric and pancreatic tumors but reduces aggressiveness in melanoma [31].

In conclusion, our results showed that both TET3 and OGT are involved in the regulation of *FOXC1*, *TWIST1*, and *ZEB1* in endometrial cancer and affect cancer cell migration and invasion. These results contribute to the knowledge of the complex mechanism supporting endometrial cancer progression; however, further studies are needed to elucidate the significance of particular EMT inducers in endometrial cancer progression.

## 4. Materials and Methods

### 4.1. Patients and Tissue Samples

Samples of endometrial cancer were obtained from 131 patients who underwent surgery in the Department of Gynecological Oncology Copernicus Memorial Hospital (Łódź, Poland). Tissue samples after tumor resection were immediately placed in RNAlater (Ambion®, Carlsbad, CA, USA). Samples were subsequently stored at −80 °C until RNA and DNA extraction. All cancer samples were characterized in terms of tumor stage according to the International Federation of Gynecology and Obstetrics (FIGO) criteria, histological grade, and type, according to WHO classification, and the ability of cancer cells to metastasize to lymph nodes. The investigations were approved by the Bioethical Commissions of the University of Lodz (6/KBBN-UŁ/III/2014).

### 4.2. Cell Culture and Treatment

Endometrial cancer cell line HEC-1A was obtained from the American Type Culture Collection (Manassas, VA, USA) and Ishikawa cells were obtained from the European Collection of Authenticated Cell Cultures (Wiltshire, UK). Cells were cultured in DMEM: F12 media (Lonza, Basel, Switzerland) containing 10% (HEC-1A) or 5% (Ishikawa) (*v/v*) FBS at 37 °C and 5% CO_2_.

Overexpression of TET3 and OGT was established by transfection of FH-TET3-pEF (#49446, Addgene, Watertown, MA, USA) or pCMV6-OGT-myc (#RC224481, OriGene, Rockville, MD, USA) into cells with Lipofectamine™2000 (Invitrogen^TM^, ThermoFisher Scientific, Grand Island, NY, USA). For controls, empty vectors were used. Knockdown experiments were performed using Silencer Select siRNA (ID: s47238 ID: s16093) (Ambion^®^, Carlsbad, CA, USA). To knockdown TET3 or OGT siRNA targeting both genes were complexed to Lipofectamine RNAiMAX (Invitrogen^TM^, ThermoFisher Scientific, Grand Island, NY, USA) following the manufacturer’s specifications. The siRNAs were used at a concentration of 30 nM. Cotransfection was performed with Lipofectamine™ 2000 (Invitrogen^TM^, ThermoFisher Scientific, Grand Island, NY, USA). Similar to single transfection in co-transfection experiments, the concentration of siRNA was 30 nM and the ratio of DNA to Lipofectamine was 1:2. The effect was analyzed 24 h after transfection.

### 4.3. RNA Isolation and RT-PCR

Total RNA from cancer tissue samples was isolated using Trizol^®^ Reagent (Sigma Aldrich, Saint Louis, MO, USA) and from cells using the ExtractMe Total RNA Kit (Blirt, Gdańsk Poland) according to the manufacturer’s instructions. First-strand cDNAs were obtained by reverse transcription of 2 μg of total RNA using High Capacity cDNA Reverse Transcription Kit (ThermoFisher Scientific, Waltham, MA, USA) following the manufacturer’s protocol. Real-time amplification of the cDNA was performed using TaqMan^®^ Gene Expression Assay (ThermoFisher Scientific, Waltham, MA, USA) according to the manufacturer’s instructions. The fluorogenic, FAM-labeled probes, and the sequence-specific primers for TET3, OGT, FOXA1, FOXC1, TWIST1, and ZEB1, and the internal control HPRT1 were obtained as inventoried assays: Hs00379125_m1, Hs01023894_m1, Hs00559473_s1, Hs01379963_m1, Hs01379963_m1, and Hs02800695_m1 (Applied Biosystems, ThermoFisher Scientific, Waltham, MA, USA). PCR reactions were carried out using the Mastercycler ep realplex (Eppendorf, Hamburg, Germany). The equation 1000∗2^−ΔCt^ was applied to calculate the expression of studied genes in tissue samples, where ΔCt = Ct of the target gene − Ct the reference gene (HPRT1). Results are expressed as a number of target gene mRNA copies per 1000 copies of HPRT1 mRNA. Fold differences in genes expression in cells normalized to HPRT1 levels were calculated using the formula 2^ΔΔCt^.

### 4.4. Isolation of Cytoplasmic, Nucleoplasmic, and Chromatin Fractions

Cytoplasmic, nucleoplasmic, and chromatin fractions were prepared from a pellet of cultured cells according to Yu et al. [32]. Cells were resuspended in lysing buffer (10 mM HEPES (ang. (4-(2-hydroxyethyl)-1-piperazineethanesulfonic acid)) pH 7.4, 10 mM KCl, 0.05% Triton X-100). After centrifugation at 14,000 rpm (4C), 10 min supernatant containing cytoplasmic proteins was moved to new Eppendorf probes and pellet containing nuclear proteins after washing with lysing buffer was resuspended in a low salt buffer (10 mM TrisHCl pH 7.4, 0.2 mM MgCl_2_, 1% Triton X100). After centrifugation at 14,000 rpm for 10 min, supernatant containing nucleoplasmic proteins was moved to new Eppendorf probes and pellet with chromatin proteins was resuspended in 0.2 M HCl. Supernatant obtained after centrifugation contained chromatin proteins.

### 4.5. Chromatin Immunoprecipitation 

Chromatin immunoprecipitation (ChIP) analysis was performed for HEC-1A and Ishikawa endometrial cancer cells treated with plasmid DNA or siRNA. Following treatment, cells were cross-linked for 10 min with formaldehyde at room temperature; the cross-linking was stopped by adding glycine at a final concentration of 125 mM. Cells were washed and lysed in lysing buffer (10 mM HEPES, 85 mM KCL, 0.5% Triton X-100, 1 mM PMSF). After centrifugation (700 rpm, 4 °C, 2 min), nuclei in pellet were resuspended in high salt buffer and sonicated on ice using Vibra Cell TM model VCX-130 (Sonics & Materials Inc. Newtown, CT, USA). The chromatin fragments were then immunoprecipitated with specific antibodies. The following antibodies were used: anti-OGT (#5368; Cell Signaling Technology), anti-TET3 [C3] C-term (GTX121453, GeneTex Irvine, CA, USA), anti-Histone H2B (glcnac S112) (ab130951; Abcam, Cambridge, UK), anti-Ubiquityl-Histone H2B (Lys120) (D11) XP^®^ (#5546, Cell Signaling Technology, Danvers, MA, USA), and anti-Tri-Methyl-Histone H3 (Lys4) (C42D8) (#9751, Cell Signaling Technology, Danvers, MA, USA). A control immunoprecipitation using IgG was set up in parallel to distinguish nonspecific precipitation. The anti-Normal Rabbit IgG (#2729, Cell Signaling Technology, Danvers, MA, USA) antibody was used. Protein A/G Plus agarose beads were used to bind protein–antibody complexes. Differential chromatin enrichment was quantified using real-time quantitative PCR. The primer pairs were: for FOXC1 forward ATGGCGATTTGATTACAGAC and reverse ATTACTGCTTAAGTGTTGCC, for TWIST1 forward CTAGATGTCATTGTTTCCAGAG and reverse CCCTGTTTCTTTGAATTTGG, for ZEB1 forward AAAGATGATGAATGCGAGTC and reverse TCCATTTTCATCATGACCAC. The results were calculated as % input (% input = % input of specific antibodies probes − % input mock IgG) and are presented as a fold change (FC = % input of treated cells sample/% input of control cells sample).

### 4.6. Western Blotting 

Endometrial cancer cells were lysed in a RIPA buffer (50 mM Tris HCl pH 8, 150 mM NaCl, 1% Nonidet P-40, 0.5% sodium deoxycholate, 0.1% SDS, 1 mM EDTA, 1 mM PMSF). Concentrations of protein were determined using the Lowry method. Proteins of the cell lysates were resolved by 8% SDS-PAGE and transferred to Immobilon P membranes. The blots were incubated for two hours at room temperature with the following primary antibodies: anti-OGT (#5368) (diluted 1:2000, Cell Signaling Technology, Danvers, MA, USA), anti-lamin A/C (sc-376248) (diluted 1:2000; Santa Cruz Biotechnology, Dallas, TX, USA), anti-Histone H3 (ab1791) (diluted 1:2500; Abcam), and anti-β-actin (sc-4778) (diluted 1:5000; Santa Cruz Biotechnology, Dallas, TX, USA). After washing with TBST (Tris buffered saline with Tween-20), immunoblots were incubated 1h at room temperature with goat anti-mouse or anti-rabbit secondary antibodies conjugated with horseradish peroxidase (diluted 1:5000, Cell Signaling Technology, Danvers, MA, USA).

### 4.7. Migration/Invasion Assay

The Transwell assay was performed to assess the rate of migration or invasion of HEC-1A and Ishikawa cells after treatment. Cell culture inserts Millicell™ (polyethylene terephthalate PET membranes with 8 μm pores) (Merck Millipore, Burlington, MA, USA) were used. Cells were plated in serum-free medium 24 h after treatment and placed in the upper chamber. The lower chamber was filled with serum-containing medium. Cells were cultured for 24 h. After that, cells in the upper chamber were removed, and migrated cells at the bottom of the inserts were fixed in 4% paraformaldehyde and stained with Giemsa. In case of invasion, assay chambers were coated with Matrigel^®^ Matrix Basement Membrane (Corning, New York, NY, USA).

### 4.8. Statistical Analysis

Differences between the expression levels of genes among the studied tissue sample groups were analyzed using the nonparametric Kruskal-Wallis test with the post-hoc Dunn test. Correlations between different gene expressions were analyzed using the Spearman test. The Student’s paired *t*-test was used to compare the differences between treated and control cells. A *p*-value < 0.05 was considered to indicate a statistically significant difference.

## Supplementary Materials

The following are available online at https://www.mdpi.com/article/10.3390/ijms222413239/s1.
