# Supplementary material for: TET3- and OGT-Dependent Expression of Genes Involved in Epithelial-Mesenchymal Transition in Endometrial Cancer
Source: Int J Mol Sci. 2021 Dec 8;22(24):13239. doi: 10.3390/ijms222413239 (PMC8708691; doi:10.3390/ijms222413239)
Supplement: Supplementary file 1 [file ijms-22-13239-s001.zip › ijms-1469382-supplementary.pdf]

Supplementary materials

TET3 and OGT dependent expression of genes involved in epithelial-mesenchymal transition in endometrial cancer

Ciesielski P, Jóźwiak P, Forma E, Krześlak A

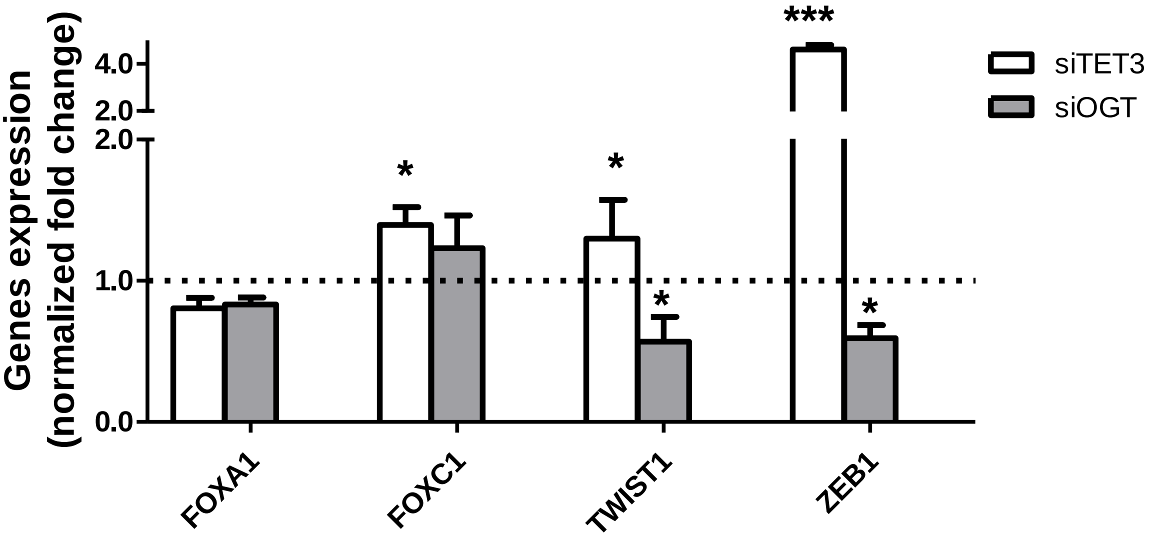

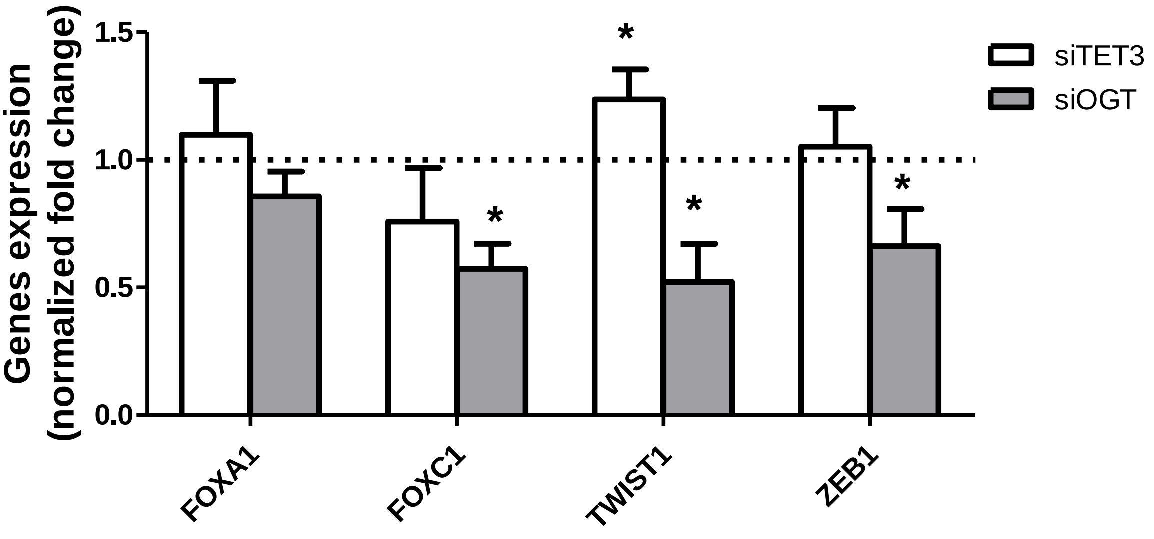

Figure S1. Expression of genes in HEC-1A and Ishikawa cells with TET3 or OGT downregulation . The mRNA levels were analyzed by Real time PCR method. The expression of genes in each kind of samples were compared to controls, i.e. cells treated with non-silent

siRNA duplexes in which expression was assumed to be 1. Data show mean ± SE(n=5), $^*p<0.01$, $^{**}p<0.001$

Table S1. Characteristics of patients and endometrial cancer samples

**Characteristic**	**Number of patients**
Patients age (63.54±10.16)	131
FIGO stage	
I	87
II	15
III	25
IV	4
Histological grade	
G1	29
G2	81
G3	21
Lymph node metastasis	
Yes	112
No	19

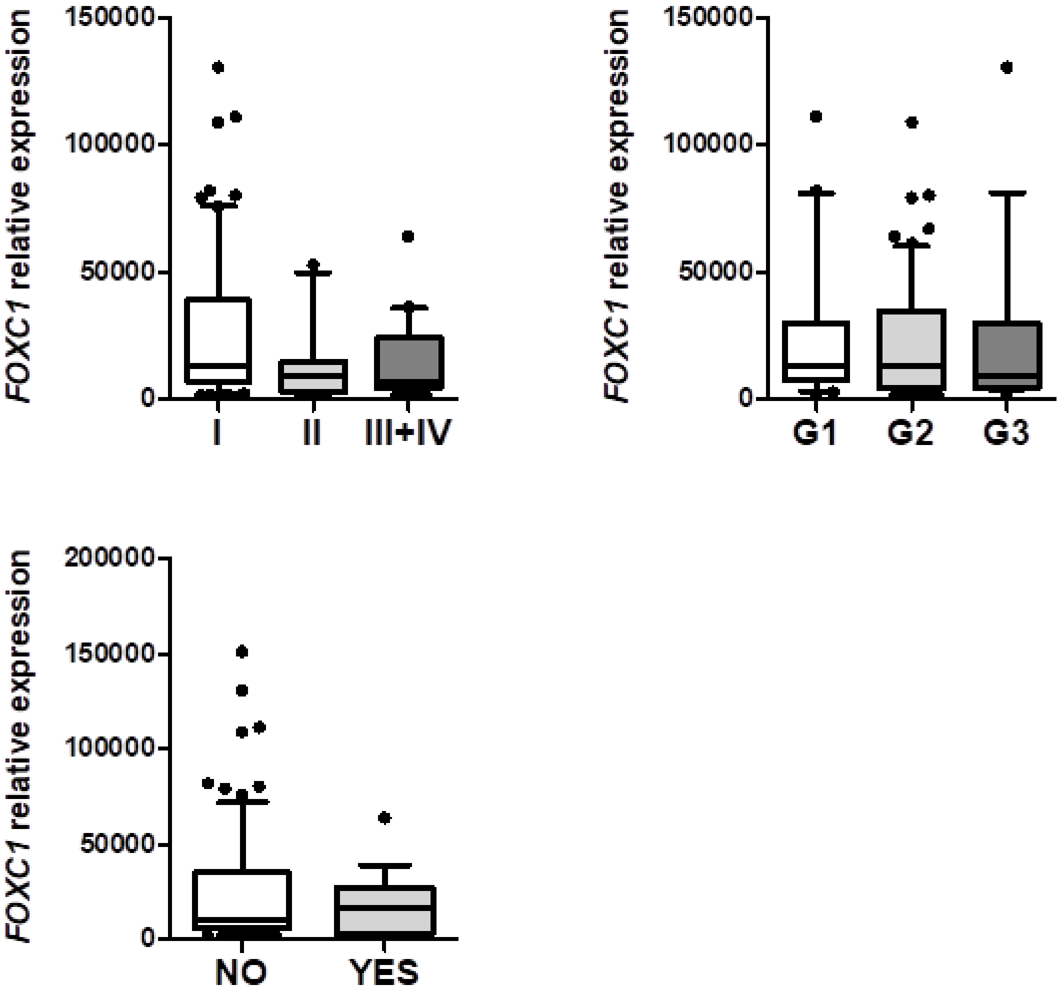

Figure S2. Expression of FOXC1 mean mRNA measured by real-time PCR in endometrial cancer samples; a comparison between subgroups with different FIGO stage, , histological differentiation grade, and lymph node metastasis status. The results are shown as number of each FOXC1 mRNA copies per 1000 copies of HPRT1 mRNA (reference gene). Graphs represent mean ± SEM. The number of cases in each group is shown in Table S1

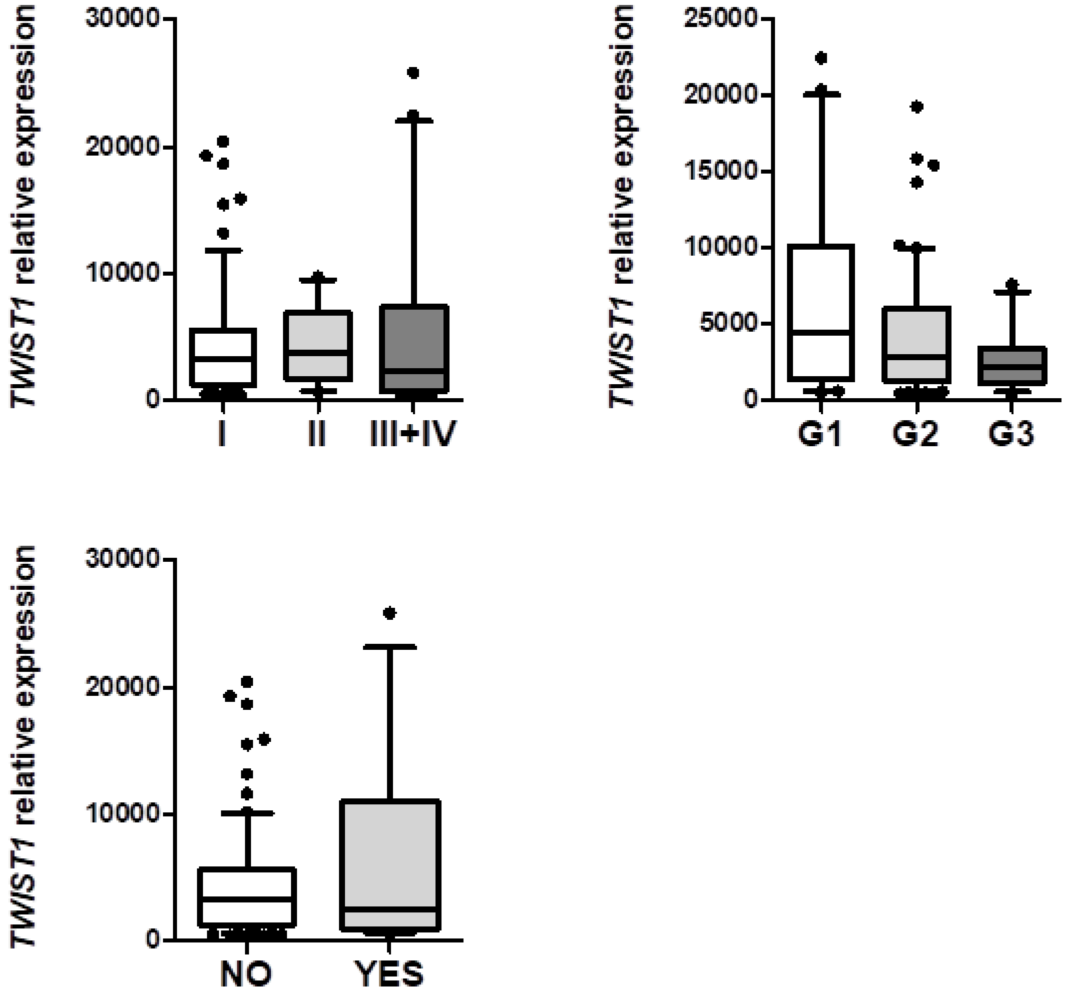

Figure S3. Expression of TWIST1 mean mRNA measured by real-time PCR in endometrial cancer samples; a comparison between subgroups with different FIGO stage, , histological differentiation grade, and lymph node metastasis status. The results are shown as number of each TWIST1 mRNA copies per 1000 copies of HPRT1 mRNA (reference gene). Graphs represent mean ± SEM. The number of cases in each group is shown in Table S1

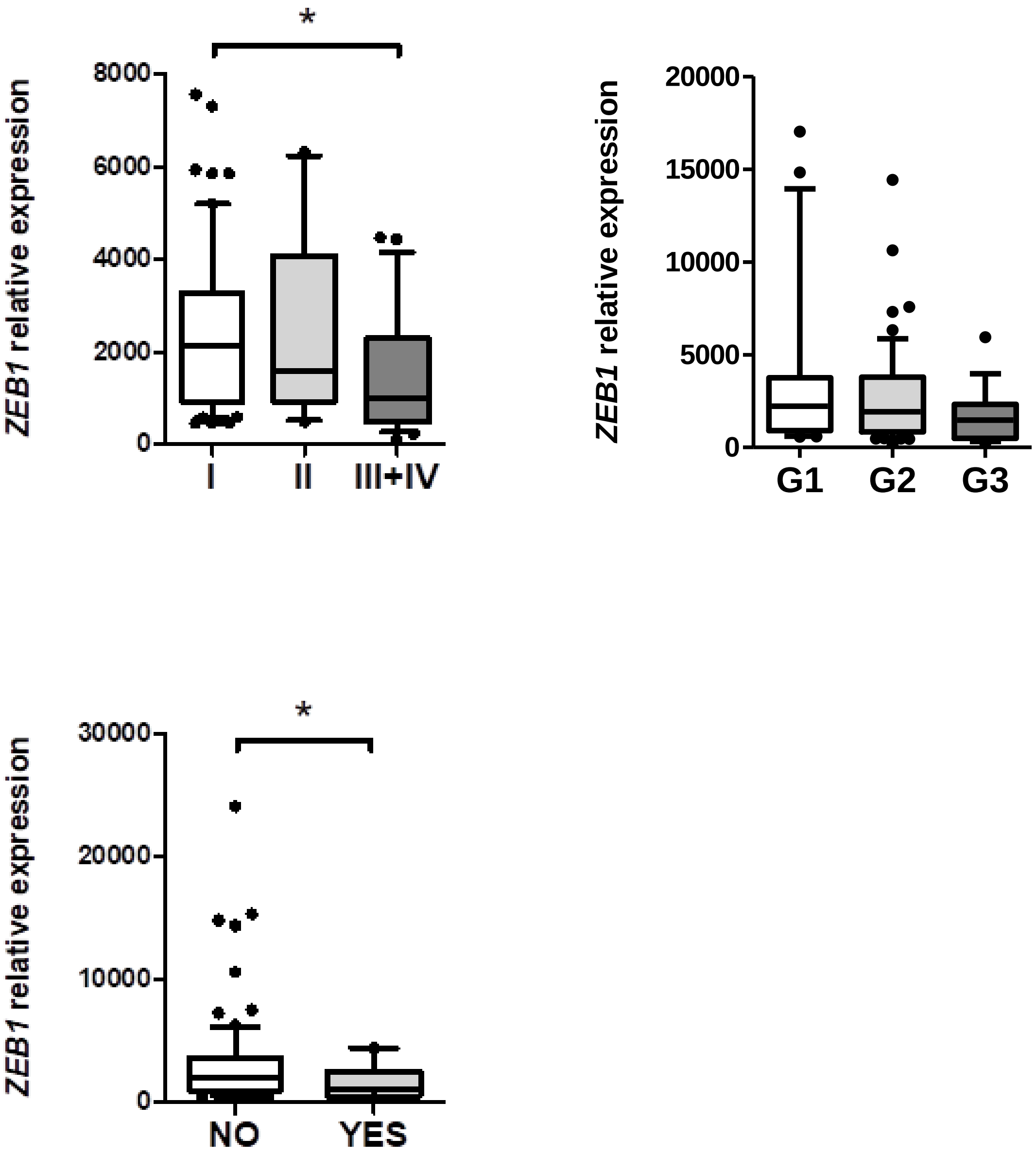

Figure S4. Expression of ZEB1 mean mRNA measured by real-time PCR in endometrial cancer samples; a comparison between subgroups with different FIGO stage, histological differentiation grade, and lymph node metastasis status. The results are shown as number of each ZEB1 mRNA copies per 1000 copies of HPRT1 mRNA (reference gene). Graphs represent mean ± SEM. The number of cases in each group is shown in Table S1.
